# Comparative performance of lateral flow immunochromatography, iELISA and Rose Bengal tests for the diagnosis of cattle, sheep, goat and swine brucellosis

**DOI:** 10.1371/journal.pntd.0007509

**Published:** 2019-06-19

**Authors:** Amahyel M. Gusi, Wilson J. Bertu, M. Jesús de Miguel, Lucía Dieste-Pérez, Henk L. Smits, Reuben A. Ocholi, José M. Blasco, Ignacio Moriyón, Pilar M. Muñoz

**Affiliations:** 1 Brucellosis Research Unit, National Veterinary Research Institute, Vom, Plateau State, Nigeria; 2 Unidad de Producción y Sanidad Animal, Instituto Agroalimentario de Aragón (IA2), Centro de Investigación y Tecnología Agroalimentaria de Aragón (CITA)-Universidad de Zaragoza, Zaragoza, Spain; 3 KIT Biomedical Research, Royal Tropical Institute / Koninklijk Instituut voor de Tropen (KIT), Amsterdam, The Netherlands; 4 Departamento de Microbiología y Parasitología, Instituto de Salud Tropical e Instituto de Investigación Sanitaria de Navarra (IdISNA), Universidad de Navarra, Pamplona, Spain; Baylor College of Medicine, UNITED STATES

## Abstract

**Background:**

Brucellosis is a world-wide extended zoonosis that causes a grave problem in developing economies. Animal vaccination and diagnosis are essential to control brucellosis, and the need for accurate but also simple and low-cost tests that can be implemented in low-infrastructure laboratories has been emphasized.

**Methodology:**

We evaluated bovine, sheep, goat and swine lateral flow immunochromatography assay kits (LFA), the Rose Bengal test (RBT) and a well-validated protein G indirect ELISA (iELISA) using sera of *Brucella* culture-positive and unvaccinated brucellosis free livestock. Sera from cattle vaccinated with S19 and RB51 brucellosis vaccines were also tested. Finally, we compared RBT and LFA using sera of white Fulani cattle of unknown bacteriological status from a brucellosis endemic area of Nigeria.

**Results and conclusions:**

Although differences were not statistically significant, RBT showed the highest values for diagnostic sensitivity/specificity in cattle (LFA, 96.6/98.8; RBT, 98.9/100; and iELISA, 96.6/100) and the iELISA yielded highest values in sheep (LFA, 94.0/100; RBT, 92.0/100; iELISA, 100/100), goats (LFA, 95.7/96.2; RBT, 97.8/100; iELISA, 100/100) and pigs (LFA, 92.3/100; RBT, 92.3/100; iELISA, 100/100). Vaccine S19 administered subcutaneously interfered in all tests but conjunctival application minimized the problem. Although designed not to interfere in serodiagnosis, vaccine RB51 interfered in LFA and iELISA but not in the RBT. We found closely similar apparent prevalence results when testing the Nigerian Fulani cattle by RBT and LFA. Although both RBT and LFA (showing similar diagnostic performance) are suitable for small laboratories in resource-limited areas, RBT has the advantage that a single reagent is useful in all animal species. Considering these advantages, its low cost and that it is also useful for human brucellosis diagnosis, RBT might be a good choice for resource-limited laboratories.

## Author summary

Brucellosis is an important zoonosis of worldwide distribution with a heavy impact wherever domestic livestock are bred, including extensive areas of developing economies. The diagnosis of brucellosis is hampered by the absence of pathognomonic symptoms, and thus accurate laboratory tests are essential. Many serological tests have been proposed but most of them are technically sophisticated and expensive and, therefore, unsuitable for laboratories in resource-limited areas. The need for simple and inexpensive tests has been expressed continuously in works dealing with brucellosis in Africa. We present an evaluation of two simple tests, the lateral flow immunochromatography assay (LFA) and the Rose Bengal test (RBT), carried out with gold standard sera (i.e, sera from *Brucella* culture-positive and brucellosis-free unvaccinated animals) from cattle, sheep, goats and swine, in comparison with an indirect ELISA (iELISA). We also performed an evaluation in cattle vaccinated with S19 and RB51 brucellosis vaccines. Moreover, we compared RBT and LFA for assessing the apparent prevalence of brucellosis in cattle in an endemic area of Nigeria. Our results showed similar diagnostic sensitivity and specificity for the three tests and disproved the extended misconception that rough brucellosis vaccines do not to interfere in serodiagnosis and that, therefore, are optimal tools for controlling the disease in resource-limited areas. Considering their diagnostic performance and simplicity, we conclude that both RBT and LFA are suitable for laboratories in resource-limited areas. RBT has the additional advantage of its low cost and usefulness for the diagnosis of human brucellosis.

## Introduction

Brucellosis is a highly contagious zoonotic disease caused by bacteria of the genus *Brucella*. Cattle, small ruminants and swine are the preferred hosts of *B*. *abortus*, *B*. *melitensis* and *B*. *suis*, respectively, and the disease causes abortions and infertility in these animals, all of which are the most common source of human brucellosis, a grave and debilitating disease. Brucellosis has a worldwide distribution and is consistently ranked among the most economically important zoonosis affecting developing economies [1]. Its control requires vaccination of domestic ruminants and a correct diagnosis but the lack of specific symptoms makes laboratory tests strictly necessary. When laboratory facilities are scanty, a common situation in endemic areas, such tests should be inexpensive, simple and robust [2]. Whereas bacteriological methods are cumbersome, require high skills and appropriate facilities, indirect tests that detect antibodies to the *Brucella* O-polysaccharide of the smooth lipopolysaccharide (S-LPS) have found wide application [3].

The lateral flow immunochromatography assay (LFA) is a rapid diagnostic test originally developed for the detection of IgM and IgG specific for *Brucella* S-LPS in human sera [4–7] that has been modified to detect anti-S-LPS IgG of bovines, sheep, goats or pigs (see Material and Methods for a detailed description of the kits). Using a Bayesian approach, Bronsvoort et al. [8] studied a bovine-LFA using a competitive ELISA as a reference in African Zebu cattle of unknown brucellosis status. These authors concluded that this LFA was very sensitive and specific (c.a. 87% and 97%, respectively) and recommended LFA over RBT on the assumption that the latter lacks specificity. Ashraf et. al [9]examined sera from sheep (n 55) and goats (n 45) of unknown individual brucellosis status and found close parallelism between LFA used (presumably species specific) and RBT. Shome et al. [10] also found parallelism between RBT and an in-house developed bovine-LFA in the sera of 153 buffaloes of unknown individual brucellosis status using an indirect ELISA (iELISA) as the reference. Manasa et al. [11] investigated a protein G-based LFA using sera of cattle (including buffaloes), small ruminants and pigs of unknown brucellosis status and also reported similar results for RBT and LFA. On the other hand, discrepancies between RBT and bovine-LFA and low relative specificity or sensitivity, respectively, have been reported in two studies with sera (n = 40 in both) of cattle of unknown brucellosis status using an iELISA as the reference [12, 13]. However, none of these studies used sera from *Brucella*-infected animals defined by a thorough bacteriological search and unvaccinated animals from brucellosis free areas, the unquestionable positive and negative gold standards that are required for the proper evaluation of brucellosis serological tests [14]. Abdoel et al. [15] found 90%, 100% and 90% positive results in bovine-, goat- and sheep-LFA in 10 cattle, 8 goats and 12 sheep, respectively, all of them with proven infection by *B*. *melitensis*. Similarly, these authors reported 75% sensitivity in 10 *B*. *suis* culture-positive pigs. Discrepancies, however, were found by these authors when comparing the LFA results with those of RBT and complement fixation using larger numbers of animals of unknown bacteriological status. No study has investigated the interference of brucellosis vaccination in the LFA.

Considering the limitations in methodology and/or number of samples of the above-summarized LFA studies, the contradictory results reported by some authors and the interest of this test for resource-limited settings, we investigated the diagnostic specificity (DSp) and sensitivity (DSe) of LFA, RBT and a multispecies protein G iELISA [16] using gold standard sera of livestock naturally infected with *B*. *abortus*, *B*. *melitensis* or *B*. *suis*, of animals from brucellosis-free areas and of vaccinated cattle. Since LFA would be particularly useful wherever laboratory facilities are limited, we also compared its performance with that of RBT using sera from infected herds of an endemic area in Africa [17].

## Methods

### Serological tests

RBT was performed according to [4] with an antigenic suspension obtained from the Laboratorio Nacional de Referencia para la Brucelosis (Granada, Spain). The indirect ELISA (iELISA) used and its standardization for diagnosis with a panel of sera from culture-positive and *Brucellosis-free* animals has been described in detail in a previous work [16]. Briefly, 100 μL of appropriate serum dilutions in 0.05% Tween 20 in 10 mM phosphate-buffered saline (pH 7.2) were added to duplicate wells of *B*. *melitensis* S-LPS-coated plates, and the plates incubated for 45 min. at 37°C. After washing, bound antibodies were detected with recombinant protein G-peroxidase (Pierce Chemical Co., Rockford, Ill.) and 0.1% 2,2-azinobis(3-ethylbenzothiazolinesulfonic acid) diammonium salt (Sigma Chemical Co., St. Louis, Mo.) and 0.004% hydrogen peroxide in 0.05 M citrate buffer (pH 4). Optical density at 405 nm was measured (Multiskan RC; Thermo Labsystems, Vantaa, Finland) after 15 min. at room temperature. Duplicate tests of negative and positive control sera were repeated in each plate as controls, and the results were expressed as percentages of average optical density with respect to the average optical density of the positive control serum. LFA kits were kindly provided by Life Assay Diagnostics Ltd (Cape Town, South-Africa). Each kit is a plastic device containing a nitrocellulose strip flanked at one end by a sample pad adjacent to a reagent pad and by an absorption pad at the other end. The detection zone has a test line with crude *B*. *abortus* S-LPS and a control line with bovine, goat, ovine or swine IgG. Detection reagents for the LFA consist of colloidal gold conjugates of affinity-purified antibodies against bovine, goat, ovine or swine immunoglobulins. The tests were performed by addition of 5 μL of serum to the sample pad followed by 130 μL of running fluid (1.67% bovine serum albumin, 3% Tween 20 in phosphate buffered saline [pH 7.6]). Test results are read after 10 min by visual inspection for staining of the test and control lines.

Both RBT and LFA reactions were read always independently by at least 2 different technicians unaware of the expected results.

We calculated the diagnostic sensitivity (Dse = 100 x Number of positive results / Number of *Brucella* culture-positive sera tested) and specificity (Dsp = 100 x Number of negative results / Number of *Brucellosis-free* sera tested) for each test using the gold standard sera collections described below. We compared also the percentage of positive animals (apparent prevalence) obtained with LFA and RBT in Nigerian unvaccinated Fulani herds.

Additional information of the tests used is provided in Table 1, where approximate costs and other technical features of each test can be found.

**Table 1:** Costs and technical features of the tests used (RBT, LFA and ELISA).

Features	RBT	LFA	ELISA	More advantageous tests according to this feature
Cost ($) of reagents or commercial kits per sample ^1^	0.20–0.50	4–6 ^2^	3–8 ^2^	RBT
Need for specific equipment ^3^	NO	NO	YES ^4^	RBT & LFA
Technical difficulty	Low	Low	Medium-High	RBT & LFA
Time required to process 100 to 1000 samples (h)	0.3 to 3	0.3 to 3	3 to 4	RBT & LFA
Automated reading of results	NO	NO	YES	ELISA
Need for serum pre-dilution	NO	NO	YES	RBT & LFA
Suitable for highly haemolyzed serum	NO	YES	YES	LFA & ELISA
Immunoglobulin detected	IgM and IgG	IgG	IgG ^5^	RBT
Useful for human diagnosis	YES	NO	NO	RBT

^1^ Calculated as the division of the cost (approx.) of the material, reagents or commercial kits required by the number of samples tested.

^2^ Based on commercial kits prices found for different providers and countries.

^3^ Small common equipment like pipettes or washing devices not considered.

^4^ Absorbance microplate reader (cost from 5.000 to 10.000 $).

^5^ It depends on the conjugate used: the “in house” ELISA used in this work and most commercial ELISA kits detect IgG exclusively but ELISA tests detecting both IgM and IgG are also available

### Sera

The following groups of sera were used:

Cattle: (i), eighty-eight naturally infected cows from which *B*. *abortus* biovar (bv) 1 or 3 (n = 59) or *B*. *melitensis* bv 3 (n = 29) had been isolated; (ii), eighty-four cows from an unvaccinated brucellosis-free herd with no history of the disease in the past 20 years; (iii), eleven brucellosis-free cows vaccinated subcutaneously with *B*. *abortus* S19 (10 x 10^10^ CFU/animal) bled 10 and 21 weeks after vaccination; (iv), twenty-two brucellosis-free cows vaccinated by conjunctival route with *B*. *abortus* S19 (5 x 10^9^ CFU/animal) bled 10 weeks after vaccination; and (v), twenty-two brucellosis-free cows vaccinated subcutaneously with the R vaccine strain *B*. *abortus* RB51 (35 x 10^9^ CFU/animal) bled between 9 and 18 months after vaccination; and (vi), a total of 178 unvaccinated cows from adult nomadic Fulani herds from various areas endemic for brucellosis in Nigeria.

Sheep: (i), one hundred naturally-infected animals from which *B*. *melitensis* bv 1 or 3 had been isolated; (ii), one hundred and one animals from an unvaccinated brucellosis-free flock with no history of the disease in the past 20 years.

Goats: (i), forty-six naturally infected animals from which *B*. *melitensis* bv 1 or 3 had been isolated; (ii), fifty-two animals from an unvaccinated brucellosis-free flock with no history of the disease in the past 20 years.

Swine: (i) thirty-nine naturally infected domestic pigs from which *B*. *suis* bv 2 had been isolated; (ii), forty-six animals from a brucellosis-free farm.

Sheep, goat and pig sera and cattle sera from groups (i) to (v) were from the collection of CITA—Unidad de Sanidad Animal (Zaragoza, Spain). Their origin and, where appropriate, the bacteriological procedures used in the isolation of *Brucella* have been described in previous works [18–21]. Cattle sera of group (vi) belong to the collection of field sera kept at the National Veterinary Research Institute of Nigeria (Vom, Plateau state, Nigeria) and were taken from unvaccinated Fulani herds in areas where *B*. *abortus* bv 3 was consistently isolated [17].

## Results

There were no discrepancies between technicians when reading reactions. As can be seen in Table 2, LFA, RBT and iELISA yielded very close results with the sera of culture-positive and brucellosis-free animals. Although differences among tests were not statistically significant (notice the overlapping Confidence Intervals in Table 2), in cattle, RBT showed a slightly higher DSe value than iELISA and LFA, and RBT and iELISA showed marginally better DSp values than LFA. In sheep, iELISA was showing the optimal diagnostic performance, followed closely by LFA and then by RBT. In goats, iELISA ranked first followed closely by RBT, being LFA the one with the lowest DSe. Likewise, iELISA showed the highest value for DSe in swine, and no differences were evidenced between RBT and LFA. Although only 1 cow and 2 goats of the brucellosis-free groups were positive in LFA, this result contrasts with the 100% DSp of RBT and iELISA in all brucellosis-free animals tested.

**Table 2:** Diagnostic sensitivity (Dse) and specificity (Dsp) of LFA, RBT and iELISA assessed with sera from *Brucella* culture-positive animals and unvaccinated animals from brucellosis free areas, respectively.

	Number of positives / *Brucella* culture-positive sera tested (DSe ^1^, 95% CI)			Number of positives / Brucellosis-free sera tested (DSp ^2^, 95% CI)		
	LFA	RBT	iELISA	LFA	RBT	iELISA
Cattle	84/87 ^3^ (96.6, 90.3–99.3)	87/88 (98.9, 93.8–100)	85/88 (96.6, 90.4–99.3)	1/83 ^4^ (98.8, 93.5–100)	0/84 (100, 95.7–100)	0/84 (100, 95.7–100)
Sheep	94/100 (94.0, 87.4–97.8)	92/100 (92.0, 84.8–96.5)	100/100 (100, 96.4–100)	0/101 (100, 96.4–100)	0/101 (100, 96.4–100)	0/101 (100, 96.4–100)
Goats	44/46 (95.7, 85.2–99.5)	45/46 (97.8, 88.5–99.9)	46/46 (100, 92.3–100)	2/52 (96.2, 86.8–99.5)	0/52 (100, 93.2–100)	0/52 (100, 93.2–100)
Swine	36/39 (92.3, 79.1–98.4)	36/39 (92.3, 79.1–98.4)	39/39 (100, 91.0–100)	0/46 (100, 92.3–100)	0/46 (100, 92.3–100)	0/46 (100, 92.3–100)

^1^ DSe = 100 x Number of positives / *Brucella* culture-positive sera tested.

^2^ DSp = 100 x Number of negatives / Brucellosis-free sera tested.

^3^ One serum of the 88 culture-positive cattle failed to produce the control line in LFA.

^4^ One serum of the 84 brucellosis-free cattle failed to produce the control line in LFA.

The interference of cattle vaccination with S19 and RB51 was also assessed (Table 3). No test was fully specific (i.e., negative in 100% of animals tested) in the S19 vaccinated cows. The percentage of reactors in any test was lower in animals vaccinated conjunctivally than in those vaccinated subcutaneously. Remarkably, a large proportion of brucellosis-free cows vaccinated with *B*. *abortus* RB51 gave a positive result in both LFA and iELISA but not in RBT.

**Table 3:** Results of LFA, RBT and iELISA in sera from brucellosis-free vaccinated cattle.

	Number of positives / Number of sera tested (DSp) at the indicated bleeding times after vaccination with:			
	S19			RB51
	Subcutaneously		Conjunctivally	
Test	10 weeks	21 weeks	10 weeks	9–18 weeks
LFA	8/10 (20%)	7/10 ^1^ (30%)	5/22 (77.3%)	11/22 (50%)
RBT	8/11 (27.3%)	5/11 (54.5%)	9/22 (59.1%)	0/22 (100%)
iELISA	9/11 (18.2%)	7/11 (36.4%)	6/22 (72.7%)	17/22 (22.7%)

^1^ One serum of the 11 animals failed to produce the control line in LFA.

**Fig 1:**
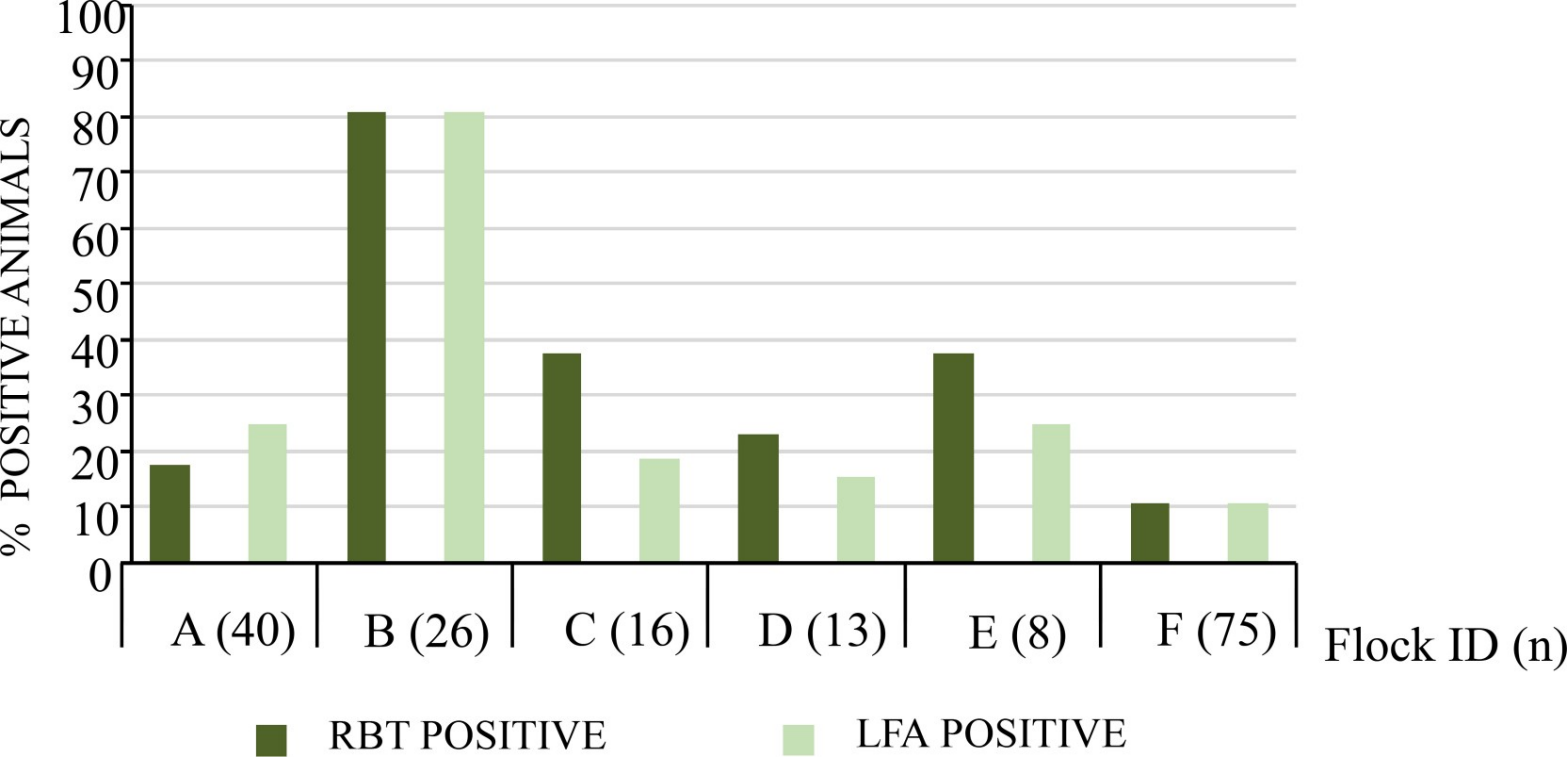
Apparent prevalence (percentage of positive animals) by RBT and LFA when testing cattle sera from a *Brucella* endemic area in Nigeria.

We also compared RBT and LFA using the sera of cattle from infected herds of an endemic area of Nigeria (Fig 1). Although both tests yielded very close apparent prevalence results, there were small discrepancies. A slightly higher number of positive sera were recorded for RBT in herds C, D and E. On the other hand, some sera in herd A were positive in LFA but not in RBT. Overall, 48 (27.0%) sera were positive by RBT while 46 (25.8%) were positive by LFA in the 178 bovine samples tested.

## Discussion

The results of this work confirm for RBT and demonstrate for LFA the good sensitivity and specificity of these simple tests. In cattle, they were not outperformed by the iELISA and this more expensive and sophisticated assay (which needs appropriate laboratory facilities and equipment -see Table 1-), yielded only slightly superior figures in small ruminants and swine. The RBT results in small ruminants, however, were not unexpected because the protocol used here was designed for the diagnosis of cattle brucellosis, and modifications to increase the proportion of serum to antigen to 3:1 (instead of 1:1 in the standard protocol) are known to increase the sensitivity of RBT in both sheep and goats [20, 22]. This suggests that the levels of serum antibodies to *Brucella* S-LPS could be lower in infected sheep and goats than in cattle, an interpretation consistent with previous observations made by iELISA [23]. Moreover, the presence of sera from recently infected animals (in which IgM response predominates) could account for the slightly highest Dse values of RBT observed in cattle, since RBT has the ability to agglutinate both IgM and IgG [3]while the LFA and iELISA here used were designed to detect IgG exclusively. However, given the small differences observed between RBT and these IgG tests, it seems that IgM detection is of little significance in the diagnosis of animal brucellosis. Like in any serological brucellosis test, the amount and quality of S-LPS antigen and conjugate must be optimized using panels of well-defined positive and negative control sera [14] and it could be that a better standardization of LFA could improve its diagnostic performance. In this regard it has to be stressed that the iELISA used here was optimized (antigen and conjugate concentrations, serum dilutions and cut-off) and validated using the gold standard serum collection (from culture-positive and brucellosis-free animals) available at CITA. These procedures account for the optimal performance of the iELISA used here [3, 14] and, therefore, caution has to be taken to draw conclusions from LFA studies compared with standardized iELISA in a different way [10–13] or with competitive ELISA [8]. The iELISA used in previous works applied cutoffs recommended by manufacturers without documented support [12] or established using the mean and standard deviation of the optical density of the populations included and/or sera of animals of unknown brucellosis status [10, 11, 13], methods that do not allow to set proper diagnostic cutoffs [14].

Our work illustrates the great parallelism existing among good *Brucella* S-LPS tests. In this context, it is worth commenting on some misconceptions on the value of simple tests. The results of this and previous extensive studies show that RBT is not prone to false negative results because of the prozone phenomenon (i.e. negative results when testing plain sera but not when diluting these). As discussed in detail in reference [3] the term prozone applied to brucellosis agglutination tests is a misnomer because it is not caused by an excess of antibody but rather by a subset of antibodies that do not agglutinate brucellae at a pH above 5. Indeed, prozones have never been observed in RBT and the likely explanation is that this test is performed at pH 3.7 [3]. Moreover, consistent with previous studies [14, 20, 22], our results show that, despite the extended misconception that RBT lacks DSp and that positive results have to be confirmed by an additional test, the DSp of RBT is optimal (100%) in ruminants in the absence of vaccination, equal or even better than that of LFA or iELISA. Therefore, it was a surprising result that 1 and 2 sera from brucellosis-free cattle and goats, respectively, developed a positive LFA reaction. Presently, we have no satisfactory explanation for this observation. The negative control sera used in this study were from brucellosis-free areas and, although bacteria like *Yersinia enterocolitica* O:9 cause false positive reactions in S-LPS brucellosis tests, such cross-reacting antibodies cannot account for the LFA false-positive results because they would have been detected also in RBT or iELISA [21]. In human brucellosis, anti-IgG autoantibodies of the IgM class (the rheumatoid factor) are a rare cause of false positive results in the IgM-LFA [24]. Therefore, although to the best of our knowledge this has not been investigated in animal serology, their presence in some sera could explain the above LFA false positive results. The close RBT-LFA parallelism in cattle sera from herds of an endemic area suggest that the specificity problem of LFA is minimal, but this should be confirmed using higher animal numbers.

*B*. *abortus* S19 and *B*. *melitensis* Rev1 are vaccines that have been instrumental in eradication programs even though they elicit antibodies reacting in all S-LPS tests, a problem considerably reduced when they are administered by conjunctival route [3, 14]. When S19 was given by this route, LFA performed as satisfactorily as the protein G iELISA used here, and both somewhat better than RBT. As an alternative, rough (R) vaccines devoid of the O-polysaccharide section of the S-LPS, on the assumption that they confer suitable protection, have been developed to eliminate the interference in S-LPS tests. However, experiments carried out under controlled conditions demonstrate that *B*. *melitensis* and *B*. *abortus* R vaccines (including RB51) are inferior to S19 or Rev 1 in protection and interfere in S-LPS iELISAs [25, 26]. RB51 is the only R vaccine that has been commercialized but claims on its usefulness for the control of the disease are based on field observations that are controversial [27–30] and inconsistent with recent retrospective analyses [31]. In addition, vaccination with RB51 does not abrogate the interference in serodiagnosis and induces protracted levels of antibodies reacting in the iELISA and LFA, although not in RBT. Although the R-LPS in RB51 lacks the S-LPS O-polysaccharide, both share the core oligosaccharide and lipid A epitopes that become accessible to antibodies upon adsorption of the antigen to a matrix, like in different ELISAs or LFA [3]. In contrast, in RBT or tests that use whole bacteria as antigen, the S-LPS inserted into the outer membrane projects the O-polysaccharide outwards hindering access of antibodies to the inner (i.e. R-LPS) epitopes [3].

In summary, both RBT and LFA are simple tests with a good performance that do not need special laboratory equipment and only require obtaining serum after clotting. While a high degree of hemolysis of the problem sera seems to be of little significance for the LFA and iELISA, it can affect the RBT performance (Table 1), a handicap to be considered and tried to overcome with suitable bleeding, transport and storage conditions. The S19 vaccine generates antibodies detected in both RBT and LFA tests and RB51 generates antibodies detected in LFA but not in RBT. In contrast to LFA that requires at least animal species specific IgG controls in the chromatographic strip even in the protein G alternative, the same RBT formulation is useful in all animal species investigated here and, moreover, it is also a very good test for the diagnosis of human brucellosis [4, 32]. Taking together these advantages and its lower cost (Table 1), RBT could be the choice in resource limited areas.

## Supporting information

S1 Checklist STARD flow diagrams and checklist. (DOCX)
