# Supplementary material for: Comparative performance of lateral flow immunochromatography, iELISA and Rose Bengal tests for the diagnosis of cattle, sheep, goat and swine brucellosis
Source: PLoS Negl Trop Dis. 2019 Jun 19;13(6):e0007509. doi: 10.1371/journal.pntd.0007509 (PMC6602290; doi:10.1371/journal.pntd.0007509)
Supplement: S1 Checklist — (DOCX) [file pntd.0007509.s001.docx]

**S1 (1). STARD flow diagram relative to the evaluation of the diagnostic performance (sensitivity and specificity) of lateral flow immunochromatography tests (LFA) for the diagnosis of cattle, sheep, goat and swine brucellosis**

Gold standard sera available

Cattle = 172; Sheep = 201; Goats = 98; Swine = 85

Sera from Brucella infected animals

Cattle = 88; Sheep = 100

Goats = 46; Swine = 39

Sera from Brucellosis free animals

Cattle = 84; Sheep = 101

Goats = 52; Swine = 46

Diagnostic specificity (95% C.I.)

Cattle = 98.8 (93.5-100); Sheep = 100 (96.4-100)

Goats = 96.2 (86.8-99.5); Swine = 100 (92.3-100)

Diagnostic sensitivity (95% C.I.)

Cattle = 96.6 (90.3-99.3); Sheep = 94.0 (87.4-97.8);

Goats = 95.7 (85.2-99.5); Swine = 92.3 (79.1-97.4)

Index test negative

Cattle = 82

Sheep = 101 Goats = 50 Swine = 46

Index test negative

Cattle = 3

Sheep = 6 Goats = 2 Swine = 3

Index test inconclusive

Cattle = 1

Sheep = 0 Goats = 0 Swine = 0

Index test inconclusive

Cattle = 1

Sheep = 0 Goats = 0 Swine = 0

Index test positive

Cattle = 1

Sheep = 0 Goats = 2 Swine = 0

Index test positive

Cattle = 84

Sheep = 94 Goats = 44 Swine = 36

**S1 (2). STARD flow diagram relative to the study of apparent prevalence by LFA and RBT in cattle sera from a *Brucella* endemic area in Nigeria**

Cattle sera available = 178

Apparent prevalence

25-28%

Reference test (RBT) inconclusive

n = 0

Index test positive

n = 46

Index test inconclusive

n = 0

Index test negative

n = 132

Reference test (RBT) positive

n = 48

Reference test (RBT) negative

n = 130

|  | **Section & Topic** | **No** | **Item** | **Reported on page #** |
| --- | --- | --- | --- | --- |
|  |  |  |  |  |
|  | **TITLE OR ABSTRACT** |  |  |  |
|  |  | **1** | Identification as a study of diagnostic accuracy using at least one measure of accuracy  (such as sensitivity, specificity, predictive values, or AUC) | #2, L 41 |
|  | **ABSTRACT** |  |  |  |
|  |  | **2** | Structured summary of study design, methods, results, and conclusions  (for specific guidance, see STARD for Abstracts) | #2, L 28-54 |
|  | **INTRODUCTION** |  |  |  |
|  |  | **3** | Scientific and clinical background, including the intended use and clinical role of the index test | #5-6, L 79-120 |
|  |  | **4** | Study objectives and hypotheses | #6, L 121-129 |
|  | **METHODS** |  |  |  |
|  | *Study design* | **5** | Whether data collection was planned before the index test and reference standard  were performed (prospective study) or after (retrospective study) | #9, L 185-191 |
|  | *Participants* | **6** | Eligibility criteria | #8-9, L 166-191 |
|  |  | **7** | On what basis potentially eligible participants were identified  (such as symptoms, results from previous tests, inclusion in registry) | #9, L 185-191 |
|  |  | **8** | Where and when potentially eligible participants were identified (setting, location and dates) | #9, L 185-191 |
|  |  | **9** | Whether participants formed a consecutive, random or convenience series | #9, L 185-191 |
|  | *Test methods* | **10a** | Index test, in sufficient detail to allow replication | #7-8, L 133-157 (proper references are given) |
|  |  | **10b** | Reference standard, in sufficient detail to allow replication | #9, L 185-191 (proper references are given) |
|  |  | **11** | Rationale for choosing the reference standard (if alternatives exist) | #6, L 110-114 |
|  |  | **12a** | Definition of and rationale for test positivity cut-offs or result categories  of the index test, distinguishing pre-specified from exploratory | #7-8, L 133-157 (proper references are given) |
|  |  | **12b** | Definition of and rationale for test positivity cut-offs or result categories  of the reference standard, distinguishing pre-specified from exploratory | #7-8, L 133-157 (proper references are given) |
|  |  | **13a** | Whether clinical information and reference standard results were available  to the performers/readers of the index test | #8, L 158-159 |
|  |  | **13b** | Whether clinical information and index test results were available  to the assessors of the reference standard | Not applicable (N.A). The Gold Standard sera collection was already available when the study was performed |
|  | *Analysis* | **14** | Methods for estimating or comparing measures of diagnostic accuracy | #8, L 160-164  #20-22, Tables 1-3 |
|  |  | **15** | How indeterminate index test or reference standard results were handled | #9, L 194  #20-22, Tables 1-3 |
|  |  | **16** | How missing data on the index test and reference standard were handled | No missing data |
|  |  | **17** | Any analyses of variability in diagnostic accuracy, distinguishing pre-specified from exploratory | N.A |
|  |  | **18** | Intended sample size and how it was determined | N.A. We used the gold standard samples available in our sera collections. |
|  | **RESULTS** |  |  |  |
|  | *Participants* | **19** | Flow of participants, using a diagram | Supplemental material |
|  |  | **20** | Baseline demographic and clinical characteristics of participants | N.A |
|  |  | **21a** | Distribution of severity of disease in those with the target condition | N.A |
|  |  | **21b** | Distribution of alternative diagnoses in those without the target condition | N.A |
|  |  | **22** | Time interval and any clinical interventions between index test and reference standard | N.A |
|  | *Test results* | **23** | Cross tabulation of the index test results (or their distribution)  by the results of the reference standard | Cross tabulation can be made easily with data presented in Table 1, #20 |
|  |  | **24** | Estimates of diagnostic accuracy and their precision (such as 95% confidence intervals) | #20, Table 1 |
|  |  | **25** | Any adverse events from performing the index test or the reference standard | #20-22, Tables 1-3 |
|  | **DISCUSSION** |  |  |  |
|  |  | **26** | Study limitations, including sources of potential bias, statistical uncertainty, and generalisability | #10-13, L 221-289 |
|  |  | **27** | Implications for practice, including the intended use and clinical role of the index test | #19, L 290-298 |
|  | **OTHER INFORMATION** |  |  |  |
|  |  | **28** | Registration number and name of registry | N.A |
|  |  | **29** | Where the full study protocol can be accessed | N.A |
|  |  | **30** | Sources of funding and other support; role of funders | #14, L 301-307 |
|  |  |  |  |  |
